# Early years interventions to improve child health and wellbeing: what works, for whom and in what circumstances? Protocol for a realist review

**DOI:** 10.1186/s13643-015-0068-5

**Published:** 2015-06-06

**Authors:** Emma Coles, Helen Cheyne, Brigid Daniel

**Affiliations:** Nursing Midwifery & Allied Health Professions Research Unit (NMAHP-RU), Scion House, University of Stirling Innovation Park, FK9 4NF Stirling, UK; School of Applied Social Science, University of Stirling, Colin Bell Building, FK9 4NF Stirling, UK

**Keywords:** Realist review, Realist synthesis, Early years, Child health, Maternal health, Child development, Wellbeing, Health inequalities, Early intervention

## Abstract

**Background:**

Child health and wellbeing is influenced by multiple factors, all of which can impact on early childhood development. Adverse early life experiences can have lasting effects across the life course, sustaining inequalities and resulting in negative consequences for the health and wellbeing of individuals and society. The potential to influence future outcomes via early intervention is widely accepted; there are numerous policy initiatives, programmes and interventions clustered around the early years theme, resulting in a broad and disparate evidence base. Existing reviews have addressed the effectiveness of early years interventions, yet there is a knowledge gap regarding the mechanisms underlying why interventions work in given contexts.

**Methods/design:**

This realist review seeks to address the question ‘what works, for whom and in what circumstances?’ in terms of early years interventions to improve child health and wellbeing. The review will be conducted following Pawson’s five-stage iterative realist methodology: (1) clarify scope, (2) search for evidence, (3) appraise primary studies and extract data, (4) synthesise evidence and draw conclusions and (5) disseminate findings. The reviewers will work with stakeholders in the early stages to refine the focus of the review, create a review framework and build programme theory. Searches for primary evidence will be conducted iteratively. Data will be extracted and tested against the programme theory. A review collaboration group will oversee the review process.

**Discussion:**

The review will demonstrate how early years interventions do or do not work in different contexts and with what outcomes and effects. Review findings will be written up following the RAMESES guidelines and will be disseminated via a report, presentations and peer-reviewed publications.

**Systematic review registration:**

PROSPERO CRD42015017832

## Background

Child health and wellbeing is influenced by multiple combining factors—physical, social, environmental, behavioural and psychological—all of which can impact on early childhood development and the acquisition of vital physical, socio-emotional and cognitive-language skills [1, 2]. Adverse early life experiences can have lasting effects across the entire life course, sustaining inequalities and resulting in negative consequences for the health and wellbeing of both individuals and society [3–6]. Babies born into families experiencing health and socio-economic disparities are at a greater risk of reduced long-term health and psycho-social outcomes and poorer quality of life; therefore, the critical pre-birth and early childhood life stages offer a unique window of opportunity to mitigate risk factors, reduce inequalities and promote lifelong health and wellbeing.

The potential to influence or even alter future outcomes via early intervention is recognised in many countries including the UK, where addressing the social determinants of health in the early years forms part of a wider commitment to improving lifelong outcomes for children and young people [7–9]. Within the four countries of the UK, there are numerous policy initiatives, programmes, interventions and strategies clustered around this theme; most originate from the national or devolved government, while others are implemented at the local level. UK public services, whether universal or targeted, are often supplemented by smaller scale interventions provided by non-profit or charitable organisations, although in recent years, public-private partnerships have become increasingly common, not only in the UK but also in Europe, Australia and Canada, where the state partners with private sector organisations to deliver services and/or provide infrastructure [10]. The proliferation of early years preventive work and associated evaluation contributes to an extensive, broad-ranging but disparate wealth of literature, resulting in an ever-increasing evidence base, albeit one with weaknesses, gaps and unanswered questions [11, 12]. This fractured evidence base can be attributed to factors such as piecemeal implementation, failure to make use of existing data, lack of theoretical frameworks to guide research and interpret data, evaluations conducted too early or with poor methodological design, attrition, lack of follow-up studies to measure medium- and long-term outcomes, difficulties replicating programmes in different contexts and a lack of appropriate and timely outcome measures that are able to reflect effectiveness and impact [2, 11, 13–15]. Measuring outcomes (intended and unintended) and linking them to the activities and processes of an intervention can present methodological challenges for evaluators, particularly in the case of complex social interventions [16].

The fact that the literature in this area appears to be disparate is one of the issues that we aim to explore in this review; it is anticipated that the gathering of relevant data is likely to support the hypothesis that the evidence base is indeed fragmented. This suggests the need to collate information on the settings and circumstances in which interventions are effective and identify the successful components that potentially are generalizable across different programmes. Dissemination of such knowledge would support a more holistic approach to the design, implementation and monitoring of early years health and wellbeing programmes, incorporating the influence of local context into the design of new interventions and the evaluation of existing programmes and taking into account the linkages between factors such as children, family, culture, community, and wider social, economic and welfare policies, in order to achieve the social change necessary to improve early childhood outcomes [2].

Many existing literature reviews in this field have focused on specific categories of early years interventions, for example, parenting, maternal and child nutrition, early years education or mental health interventions [17–21]. Other reviews have provided useful evidence summaries with regard to addressing the effectiveness of early years interventions [2, 12], thus providing a valuable contribution to the debate. Nevertheless, the question still remains, ‘what works, for whom and in what circumstances’ to improve child health and wellbeing? Further, how can this knowledge interface with current and future early years policy and practice in different jurisdictions? Existing reviews focusing on effectiveness have not tended to address the underlying causal mechanisms of why interventions do or do not work in given contexts—this review will aim to bridge that gap by providing a context-sensitive evidence synthesis focusing on exploring how and why early years interventions work.

Given the potentially broad scope of the topic area and the need to include a wider range of types of evidence than can be accommodated within a traditional systematic review framework, a realist approach [22] was deemed appropriate to answer such a broad-based research question. Realist inquiry focuses on taking an explanatory approach with an emphasis on understanding causation. It is based on the assumption that all programmes and interventions have underlying theories—whether implicit or explicit—about what might cause change and that these theories may be modified as evidence emerges [16]. Thus, it is a flexible, theory-driven method that can accommodate the complexity of interventions and the real-world relationships between the social and contextual factors and human behavioural responses that can influence the success or failure of a programme and contribute to a range of diverse outcomes and effects, whether intended or not. Despite the relative newness of the method, realist methodology has been widely used in recent health and social sciences research reviews [23–27].

### Aims of the review

The primary aims of this realist review are (i) to critically examine the impact of early years interventions on the health and wellbeing of children and (ii) to transform the wealth of data in this area into a cohesive evidence base of ‘what works, for whom and in what circumstances’, in terms of implementation in given contexts. Drawing on their previous work in maternal and infant health, child neglect/protection and realist evaluation [28–30], the reviewers will focus on synthesising available evidence to demonstrate what works to improve child wellbeing and reduce inequalities in the early years. Focusing on the period from pre-conception to the age of 5, the study will incorporate the vulnerable months of pregnancy and the immediate postnatal period when parenting skills are established and attachments formed, encompassing the vital first 1001 days of life (conception to age 2) [31, 32] as well as the transition towards school entry.

A further, secondary aim concerns the interface between research, policy and practice. After completion of the review, findings will be translated into evidence-based, practical knowledge and recommendations that can be shared with and applied by policy-makers, practitioners and service users. The scope of such dissemination is likely to be varied: one example of a practical application of findings is the proposed development of a set of short-term outcome measures, meaningful to practitioners and families, for the evaluation of the efficacy and impact of initiatives to improve wellbeing and reduce inequalities.

### Scope of the review

For the purposes of this realist review, ‘early years’ will be defined as pre-conception to 5 years of age. The definition of wellbeing will be drawn from the SHANARRI indicators [9], devised by the Scottish Government to encompass eight domains of wellbeing: safe, healthy, achieving, nurtured, active, respected, responsible and included. Inclusive of the social, physical and emotional aspects of wellbeing, the SHANARRI indicators are evidence-based [33] and are therefore generalizable to other jurisdictions with similar aspirations for children. The definition of health used here includes physical health but also includes mental health and wellbeing and the social determinants of health [34]. Accordingly, health inequalities are viewed as differences in health or health outcomes between different population groups or across the social gradient [35]; here they are defined (i) in relation to immediate negative health outcomes such as low birth weight and (ii) in terms of exposure to socio-economic risk factors—such as poverty or parental drug or alcohol misuse—that increase the possibility of or prolong poor health outcomes in the short, medium and long term [8, 36]. Evaluations of both universal services (available to all, regardless of need) and targeted programmes (based on need) will be included: large-scale or small-scale, including pre- and postnatal, interventions and programmes directed at infants and children and/or their parents/families/carers. All settings and contexts will be considered for inclusion, including but not limited to clinical, centre-based, home-based, early years educational settings and community settings. In contrast to traditional systematic reviews, this review will cross discipline and policy boundaries, drawing evidence from a broad range of international sources, and will include empirical, theoretical and experiential data. Areas of interest will include but not be limited to maternal and pre-natal/infant/child health, social care, early years education, child protection, social inclusion and public health.

### Collaboration

At the beginning of the review process, a collaboration group will be set up to oversee progress and to help develop consensus by providing a forum for monitoring, discussion and the sharing of feedback at all stages. This group will consist of the research team and members of the Scottish Midwifery Research Collaboration, a network of midwifery researchers based at various Scottish universities. The composition of the group may change or expand during the course of the review; other identified stakeholders, such as policy-makers, practitioners or service users, may be invited to collaborate with the research team as the review progresses. The group will contribute to all aspects of the review, from theory development to interpretation of findings.

This review protocol is registered on the PROSPERO database (reference number: CRD42015017832).

## Methods/design

The review will follow realist methodology, a theory-driven interpretive approach that seeks to explain the root causes of programme outcomes and diverse patterns of impacts and effects, by evaluating evidence from a range of sources [22, 37]. The aim of realist inquiry is ‘explanation building…to articulate underlying programme theories and then to interrogate the existing evidence to find out whether and where these theories are pertinent and productive’ [22]. Accordingly, the realist approach centres on the development and refinement of theory, in order to provide plausible and evidenced explanations, in descriptive narrative form, as to why and how interventions may or may not work in multiple contexts and with multiple actors [38, 39]. The principal output of a realist review, or synthesis, is an evidence-based, iteratively tested and refined ‘programme theory’—an articulation of how and why a programme or family of programmes is thought to cause its desired outcomes within various contextual configurations [40].

In realist thinking, programmes are theories [22]. The hypotheses (ideas, beliefs and intended outcomes) that lie behind each intervention or family of interventions, outlining how they are meant to work, are identified, tested and refined through the course of the realist review, with the aim of examining the extent to which these hypotheses are met in practice. Programme theories are expressed using the interlinked concepts of ‘context’ (C), ‘mechanism’ (M) and ‘outcome’ (O). Programmes attempt to alter context in some way (for example, by the provision of resources), thus affecting existing mechanisms or introducing new ones, to produce desired outcomes. Mechanisms are the responses triggered by the context, which cause outcomes, whether intended or unintended. Mechanisms will produce different outcomes in different contexts. The way in which causation occurs for any given outcome is expressed by the formula C + M = O; a programme theory may consist of multiple CMO configurations that explain the context, mechanism and outcome relationships and the pattern of outcomes. By recognising the importance of external and environmental influences, realist inquiry can be used to unpack complex programmes and interventions and explore the reasons underlying how and why they do or do not work in given contexts [41].

The review will be conducted following the five key stages of Pawson’s realist review template [22]: (1) clarify scope, (2) search for evidence, (3) appraise primary studies and extract data, (4) synthesise evidence and draw conclusions and (5) disseminate findings. The stages are presented in a linear fashion, but in practice, they are conducted iteratively with some overlap to be expected. Hence, refinements to the developing programme theory are made throughout the review process based on emerging findings.

1. Clarifying the scope of the review: exploratory searching and theory development.

As the review topic is potentially very wide-ranging, a priority at the first stage will be to refine and narrow the research question via a broad-based scoping exercise. It is at this point that key themes and concepts are defined as the focus of the review begins to take shape and the reviewers begin to develop a theoretical understanding of the research topic. Following Pawson’s methodology [22], relatively unstructured exploratory internet-based searches will be carried out, guided by a limited number of combinations of search terms (e.g. ‘early years’, ‘wellbeing’, ‘inequalities’, ‘early years intervention’), specifying the type of interventions of interest and the setting. These searches will be purposefully broad in order to locate a varied range of evidence to give an overview of the topic area. The scoping searches will be supplemented by a ‘desk drawer’ search strategy to incorporate documents and other evidence known to the review team. This initial immersion in the literature allows the project team to gain a deeper understanding of the research problem but primarily serves to facilitate the identification of, in realist terms, the underlying ‘programme theories’—in this case, explanations of how and why early years interventions are supposed to work in order to produce their desired outcomes. Therefore, the exploratory search stage forms part of an initial scoping exercise to (a) find and assemble key literature and policy documents and (b) scan these to identify and map the range of existing programme theories in order to create a framework for the review. The aim is to uncover the programme theories within the literature; these may be in the form of assumptions about how and why the programme is intended to work (i.e. the causal mechanisms), dominant themes, explicit or implicit references to theory, descriptions of the ways in which change is expected to occur as a result of the intervention or theoretical descriptions of the linkage between programme activities and outcomes.

As the scoping exercise progresses and findings begin to emerge, the reviewers will develop an outcome-focused realist programme theory, based on the identified existing programme theories and structured around CMO configurations, to explain how and why early years interventions can work, in multiple contexts, to generate outcomes that improve child health and wellbeing and reduce inequalities. In keeping with realist methodology, key stakeholders and experts, including government policy-makers, Scotland’s Commissioner for Children and Young People and representatives from non-profit/charitable children’s organisations, will be consulted throughout this first stage in order to bring in a range of additional perspectives that can contribute to the developing programme theory. Theory development will also be informed by meetings of the review collaboration group. The resulting theoretical, explanatory model and its component contexts, mechanisms and outcomes of interest will be used as the framework for the succeeding stages of the review.

(2) Search for primary evidence.

Systematic searches for primary studies that are relevant to the programme theory will then be carried out. This second search phase will involve seeking additional relevant evidence (e.g. comparative effectiveness studies, process evaluations and qualitative research), primarily for testing and refining the programme theory. In keeping with the iterative nature of realist methodology, searching will be progressively extended and refined as the review evolves. Selection of sources will be based on relevance to aspects of the programme theory. A combination of search strategies will be utilised. Internet search engine and electronic database searching will be carried using keywords based on the themes, concepts and programme theories identified in the exploratory search. This will be supplemented by a ‘cited by’ article search and a search of citations included in the reference lists of included papers. Sources of grey literature, including unpublished reports, will also be investigated. The reviewers will make use of snowballing techniques and consultation with experts and stakeholders. Given that a wide range of documents may contain evidence that can contribute to a realist review [37], multiple types of evidence will be included, including studies of any design, policy documents and surveys of participant experience of interventions. Search results from electronic databases and other sources will be imported into reference management software (RefWorks) and duplicates removed.

(3) Appraise evidence and extract data.

The realist appraisal and extraction process differs from a traditional systematic review, as inclusion and exclusion criteria are based on the programme theory and what the literature is able to contribute to it. In a realist review, the units of analysis are not the interventions themselves per se but the theories underpinning the interventions (or families of interventions). Accordingly, evidence will be selected for inclusion based on its relevance to the programme theory; the screening process will be based on an assessment of ‘fit’ to the research question, with the aim of identifying the elements of interventions that are, or could potentially be, effective. ‘Bespoke’ data extraction forms [39] will be developed incorporating key themes and questions based on the emerging findings from stage 1, designed to gather information on contexts, mechanisms and outcomes of interest, thus providing a template to interrogate the evidence. The key test for evidence in a realist review is an assessment of its relevance (whether it can contribute to theory building and/or testing) and rigour (whether the methods used to generate the evidence are credible and trustworthy) [37]. The included evidence will be appraised for relevance and rigour using a ‘fitness for purpose’ approach, following the criteria outlined in the Realist And Meta-narrative Evidence Syntheses: Evolving Standards (RAMESES group) Quality Standards for Realist Synthesis [42]. Extracted data will be put into evidence tables and organised into themes.

Again, in contrast to the traditional review process, during this stage, the project team will revisit and if necessary revise the focus of the review based on emerging findings. This may require further refinement of the inclusion/exclusion criteria and conducting further purposive searches if necessary in response to any revisions of the programme theory, followed by the integration of any additional evidence.

(4) Analysis and synthesis of evidence: test/refine theory and draw conclusions.

The key analytic process in a realist review involves iterative testing and refinement of theoretically based explanations (i.e. the programme theory) using empirical findings in data sources [37]. The goal of the fourth stage is thus to test and refine the initial programme theory by drawing comparison with the primary evidence and exploring and analysing the relationships between contexts, mechanisms and outcomes. Relevant passages of included documents will be annotated and coded to identify contexts, mechanisms, outcomes and CMO configurations. The reviewers will compare and contrast the evidence, looking for recurring patterns of CMOs across the data that are able to support, contradict or inform the, programme theory. This is an iterative process, guided by the research question and primary aims of the review. Completion of the realist synthesis will allow the reviewers to modify or refine the identified CMO configurations and use these to explain (a) how and why the interventions or families of interventions cause change and generate outcomes and (b) what works ‘for whom, in what contexts, in what respects, to what extent and over what durations’. It is at this point that overall conclusions are drawn, and a set of tentative recommendations produced. This penultimate stage will enable the production of a final synthesis integrating review evidence with programme theory and culminating in a revised programme theory, refined in light of the evidence and reflecting the review findings. Presented as a narrative, the findings will be written-up according to standards outlined by the RAMESES group [42] and will follow the format set out by the RAMESES standards.

(5) Dissemination of findings.

Findings will be translated into evidence-based, practical knowledge and recommendations that can be shared with and applied by policy-makers, practitioners and service users. They will be disseminated in the form of a final report, presentations to stakeholders and peer-reviewed publications.

## Discussion

This study will use a realist review approach to synthesise the early years intervention literature and to enable a greater understanding of ‘what works, for whom, in what circumstances and why’, in terms of preventive interventions to improve child health and wellbeing and reduce inequalities. The use of a realist approach will allow the review to demonstrate how and why early years interventions do or do not work in different contexts, by exploring the underlying programme theories and the interactions between context, mechanism and outcomes. The dissemination of findings to stakeholders and policymakers will facilitate the practical application of evidence-based concepts to improve health and wellbeing outcomes for children, parents and families.

## Abbreviations

CMO: Context, Mechanism, Outcome
RAMESES: Realist and meta-narrative evidence syntheses: evolving standards
SHANARRI: Safe, Healthy, Achieving, Nurtured, Active, Respected, Responsible, Included
